# Formation of high molecular weight p62 by CORM-3

**DOI:** 10.1371/journal.pone.0210474

**Published:** 2019-01-08

**Authors:** Toshihiko Aki, Kana Unuma, Kanako Noritake, Naho Hirayama, Takeshi Funakoshi, Koichi Uemura

**Affiliations:** Department of Forensic Medicine, Graduate School of Medical and Dental Sciences, Tokyo Medical and Dental University, Yushima, Bunkyo-ku, Tokyo, Japan; Niigata Daigaku, JAPAN

## Abstract

CORM-3 is a water-soluble carbon monoxide (CO)-releasing molecule developed for possible therapeutic use of CO. CORM-3 belongs to a group of metal carbonyl compounds that contain transition metals and carbonyls as the central scaffold and coordinated ligands, respectively. CORM-3 has been reported to be reactive with many proteins in eukaryotes including mammals. Among them, several extracellular proteins, such as lysozyme, as well as plasma albumin and fibronectin, have been shown to interact directly with CORM-3. p62 is an intracellular adaptor protein required for targeting ubiquitinated (Ub) proteins to lysosomal degradation through autophagy. p62 has been shown to undergo self-oligomerization via covalent crosslinking in response to treatment with verteporfin, a benzoporphyrin derivative used for photodynamic therapy. Here we show that CORM-3 also interacts directly with p62. When applied to mouse embryonic fibroblasts (MEFs) at a high concentration (1 mM), CORM-3 causes the formation of reduction- and detergent-resistant high molecular weight (HMW)-p62. HMW-p62 accumulates more in atg5-/- MEFs than in wild type (WT) MEFs, showing the elimination of HMW-p62 through autophagy. HMW-p62 is also generated in H9c2 rat cardiomyoblastoma as well as A549 human alveolar epithelial cells, suggesting that HMW-p62 formation is not specific to MEFs, but, rather, is a general event in mammalian cells. HMW-p62 formation by CORM-3 can be reproduced using purified p62 *in vitro*, demonstrating the direct interaction between CORM-3 and p62. These results show that p62 is a CORM-3-interactive intracellular protein.

## Introduction

CORM-3 [carbon-monoxide-releasing molecule-3, tricarbonylchloro(glycinate)ruthenium] is a transition metal (Ru)-containing carbon monoxide-releasing molecule developed for the use of carbon monoxide (CO) as therapy [1]. The inhalation of CO gas as well as the use of CORM-3 within appropriate concentrations, which are typically 100–500 ppm CO levels as well as 10–40 mg/kg body weight of CORM-3 in experimental animals, respectively, has been shown to ameliorate various diseases [2]. CO has been shown having anti-inflammatory effects through its inhibitory effect on the activation of inflammasomes [3]. Therefore, administration of CORM ameliorates inflammation-related diseases such as sepsis [4, 5] and collagen-induced arthritis [6]. There are many other disorders, such as fibrosis [7], injuries and shock after hemorrhage [8, 9] as well as ischemic lung injuries [10], that were shown to be mitigated by CO. It has also been demonstrated that 10–50 μM CORM-3 mitigates cell death caused by various stresses such as hypoxia/reoxygenation and paraquat administration [1]. However, 200–500 μM CORM-3 decreases the viability of mammalian cells [11, 12]. CORM-3 has been shown to react with several proteins including lysozyme, albumin, and transferrin [13, 14]. We have also reported recently that CORM-3, when applied at 1 mM, causes 1) the oligomerization of extracellular plasma fibronectin (FN), 2) the dissociation of fibrillar FN from the cells, and 3) resultant apoptosis in mouse embryonic fibroblasts (MEFs) [15]. Nevertheless, little about the reactivity of CORM-3 with mammalian intracellular proteins has been reported to date.

p62, also known as sequestosome1 (SQSTM1), is a protein involved in autophagy, a cellular lysosome-dependent degradation system [16]. p62 works as an adapter protein connecting ubiquitininated proteins (Ub-proteins) to the autophagosomal membrane protein LC3 [17]. Before the sequestration and degradation of Ub-protein aggregates in lysosomes, p62 undergoes self-oligomerization through non-covalent interaction. This oligomerization is necessary and sufficient for its binding to the autophagosomal membrane [18, 19]. In addition to the self-oligomerization mediated by non-covalent binding to promote autophagy, p62 also undergoes disulfide bond-mediated self-oligomerization during aging or oxidative stresses [20]. Furthermore, p62 works as an N-recognin during N-end rule-mediated protein degradation [21]. Binding of p62 to N-terminal arginylated degrons (Nt-Arg), as well as ligation of synthetic ZZ domain ligands (XIE62-1004 and XIE2008), activate p62 through facilitating disulfide bond-mediated self-oligomerization [21, 22]. In contrast to these oligomerizations, verteporfin, a compound used as therapy to treat abnormal vessel formation in the eye, has been shown causing aberrant p62 oligomerization. This verteporfin-induced high molecular weight (HMW)-p62 species are carbonylated through oxidative cellular stress and crosslinked covalently with one another through disulfide bond-independent mechanisms. This crosslinked species has less capacity for binding Ub-proteins as compared to non-crosslinked p62 and inhibit the process of autophagy [23]. Here we report that CORM-3 also induces the formation of HMW-p62, independent from disulfide-bond formation, when applied at high concentration (1 mM).

## Materials and methods

### Reagents

CORM-3 and verteporfin were purchased from Sigma-Aldrich (St Louis, MO, USA). Antibodies used were: anti-p62 (PM045, MBL, Osaka, Japan), anti-LC3 (#2775, Cell Signaling Technologies, Beverly, MA, USA), anti-cleaved caspase-3 (#9664, Cell Signaling Technologies), anti-rho-kinase-1 (ROCK-1, sc-6055, Santa Cruz Biotechnology, Santa Cruz, CA, USA), anti-fibronectin (FN, 610077, BD Biosciences, San Jose, CA, USA), anti-mono- and polyubiquitinated conjugates (FK2, BML-PW8805, Enzo Life Sciences, Farmingdale, NY, USA), and anti-actin (A2066, Sigma-Aldrich). Purified bovine plasma fibronectin (FN) (F1141, Sigma-Aldrich), recombinant human p62 (ab95320, abcam, Cambridge, UK), and bovine serum albumin (BSA) (A7906, Sigma-Aldrich) are commercially available.

### Cell culture

Wild type (WT) and atg5-/- immortalized mouse embryonic fibroblasts [MEFs, [24]] were obtained from RIKEN Cell Bank (RCB2710 and RCB2711, respectively). A549 human alveolar epithelial cells were also obtained from RIKEN Cell Bank (RCB0098) and H9c2 rat cardiomyoblastoma cells were purchased from ATCC (Manassas, VA, USA). All cells were maintained in DMEM supplemented with 10% FBS and antibiotics (100 U/ml streptomycin and 100 μg/ml penicillin) under a humidified atmosphere of air containing 5% CO_2_.

### Immunoblotting

Cell lysates were obtained by lysing cells in buffer [0.32 M sucrose, 10 mM Tris-HCl (pH 7.4), 5 mM EDTA, 50 mM NaF, 2 mM Na_3_VO_4_, and a protease inhibitor cocktail (Complete, Roche, Mannheim, Germany). Equal amounts of lysates were applied to SDS-PAGE, blotted to a PVDF membrane (Millipore, Billerica, MA, USA), and incubated with appropriate antibodies overnight at 4°C. Then, the membrane was further incubated with horseradish peroxidase (HRP)-conjugated anti-IgG (Promega, Fitchburg, WI, USA), and the antigens were visualized using a Western Lightning Chemiluminescence Reagent Plus Kit (Perkin Elmer Life Science, Waltham, MA, USA). ImageJ (1.47v) was used to quantify the band intensities.

### Immunocytochemistry

Cells grown on coverslips were fixed in 4% paraformaldehyde (PFA)/PBS, permeabilized in 0.5% Triton X-100/PBS, and incubated with appropriate 1st antibodies overnight at 4°C, followed by further incubation with Alexa488- or Alexa549- conjugated anti-IgG antibody (Molecular Probes, Eugene, OR, USA). Specimens were observed under a fluorescence microscope (Leica, DMi8, Wetzlar, Germany). For quantification of the number of p62-, Ub-, and LC3-positive dots, more than 100 dots from more than 20 cells were counted for each dot.

### In vitro HMW-species formation assay

Recombinant full-length human p62 (ab132366, abcam), purified FN from bovine plasma (F1141, Sigma-Aldrich), or bovine serum albumin (BSA) (A5611, Sigma-Aldrich) were diluted in PBS to 10 ng/μl, 10 ng/μl, or 50 ng/μl, respectively, and mixed with CORM-3, CORM-A1, RuCl_3_, or verteporfin at final concentrations of 1 mM, 1 mM, 1 mM, or 10 μM, respectively. The mixtures were then incubated at 37°C for 10–60 min, the reactions were stopped by the addition of 3xLaemmli sample buffer, and the samples were subjected to SDS-PAGE. p62 and FN were visualized by immunoblotting while BSA was visualized by CBB staining.

### Small interference RNA

Small interference RNA (siRNA) for mouse p62 (QIAGEN, SI00197239) or control siRNA (AllStars negative control siRNA, QIAGEN, SI03650318) was mixed with Lipofectamine RNAiMAX (Invitrogen, Carlsbad, CA, USA) at a final concentration of 50 nM in serum-free DMEM and exposed to MEFs for 4 h, followed by the addition of complete (serum-containing) medium and further incubation for another 44 h. Then, the cells were stimulated with CORM-3 and protein extracts were prepared and analyzed by immunoblot analysis.

### Statistical analysis

The data were evaluated by student’s *t*-test, Dunnett’s test, Tukey-Kramer’s test, or Bonferroni’s test. GraphPad Instat (Version 3.1a, GraphPad Software, Inc., La Jolla, CA, USA) was used for statistical analysis.

## Results

### CORM-3 induces apoptosis in atg5-/- MEFs

**Fig 1:**
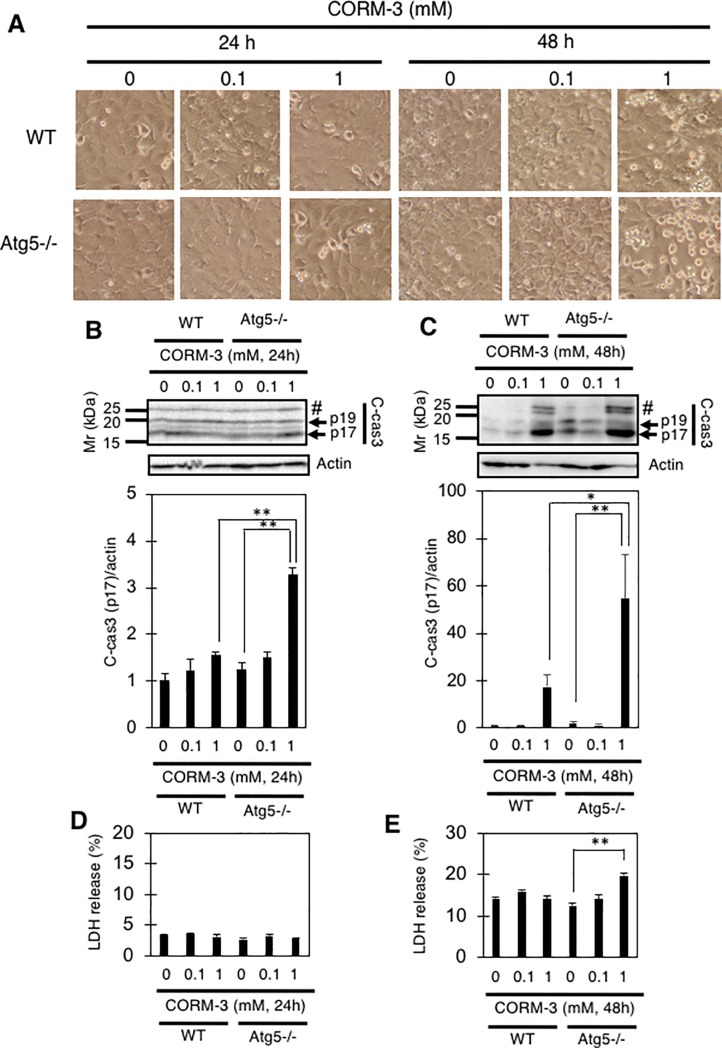
CORM-3 induces the death of MEFs. (A and B) MEFs were treated with the indicated concentrations (0, 0.1, or 1 mM) of CORM-3 for 24 or 48 hours (A), and the relative levels of p17 fragment of cleaved-caspase-3 to actin (B and C) as well as LDH release into the medium (D and E) are shown. #, uncharacterized fragment. Data represent the means and S.E. *, p<0.05; **, p<0.01 by Turkey-Kramer’s test (B and E, n = 4). *, p<0.05; **, p<0.01 by Bonferroni’s test (C, n = 3).

We have shown previously that incubation with 1 mM CORM-3 for 72 hours induces the death of wild-type (WT) MEFs [15]. Given this result, we first examined whether autophagy is involved in protecting MEFs from CORM-3-induced cell death. For this purpose, WT and atg5-/- MEFs were incubated with 0, 0.1, or 1 mM CORM-3 for 24 or 48 hours. Although no observable changes were detected in either WT or atg5-/- MEFs after 24 hours incubation with 1 mM CORM-3 (Fig 1A), a slight but significant increase in the p17 fragment of cleaved (activated)-caspase3 (c-cas3) was detected in atg5-/- MEFs as compared to WT MEFs (Fig 1B). In addition to well-characterized p17 and p19 fragments of caspase-3, we also detected an uncharacterized fragment migrating around 25 kDa and tended to increase in response to CORM-3 treatment (Fig 1B). We do not know, however, the exact identity of this fragment. After further incubation for 48 hours, appreciable morphological changes, such as cell rounding as well as detachment from the culture dish, were observed in both WT and atg5-/- MEFs incubated with 1 mM CORM-3 (Fig 1A). A significant increase in c-cas3 was observed in atg5-/- MEFs as compared to WT MEFs (Fig 1C). A slight but significant increase of LDH release was also detected in atg5-/- MEFs incubated with 1 mM CORM-3 for 48 hours (Fig 1D and 1E). These results not only confirm our previous report that 1 mM CORM-3 exerts cytotoxicity on MEFs, but also shows that autophagy is involved in protecting against the cell death caused by CORM-3.

### HMW-p62 accumulates in atg5-/- MEFs after exposure to CORM-3

**Fig 2:**
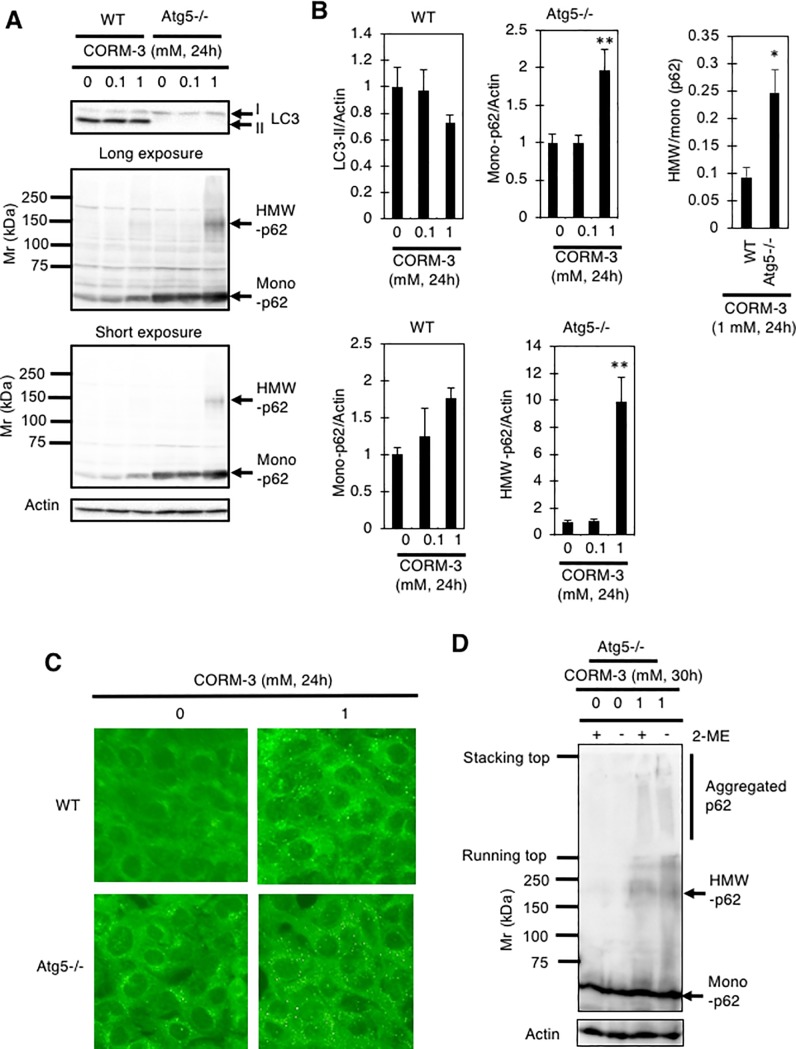
Generation of HMW-p62 by CORM-3 in MEFs. (A and B) MEFs treated with 0, 0.1, or 1 mM CORM-3 for 24 hours were subjected to immunoblot analysis using anti-LC3 and -p62 antibodies. Relative levels of LC3-II, as well as HMW- and mono-p62, to actin are shown as means and S.E. (n = 4). **, p<0.01 versus 0 mM by Dunnett’s test. (C) p62 dot formation in WT and atg5-/- MEFs treated with or without 1 mM CORM-3 for 24 hours. Immunofluorescence analysis was performed using anti-p62 antibody and Alexa488 (green)-labeled anti-IgG antibody. (D) Atg5-/-MEFs were treated with or without 1 mM CORM-3 for 30 hours and cell lysates were subjected to SDS-PAGE with or without the reduction by 2-mercaptoethanol (2-ME). Immunoblot analysis were performed using anti-p62 antibody.

We next examined the status of autophagy using the marker proteins LC3 and p62; an increase in autophagy activity can be observed as an increase in the LC3-II level and a decrease in the p62 level [25, 26]. As shown in Fig 2A and 2B, the LC3-II level remained constant in WT MEFs regardless of treatment with CORM-3, suggesting that CORM-3 does not activate autophagy, at least after 24 hours of treatment. In atg5-/- MEFs, LC3-II was absent and p62 was accumulated as compared to WT MEFs, confirming the deficiency of autophagy in these cells (Fig 2A). Unexpectedly, we observed a high molecular weight (HMW)-p62 species in MEFs administered CORM-3; as shown in Fig 2A, a faint band (~150 kDa,) became detectable in atg5-/- MEFs following exposure to 1 mM CORM-3 for 24 hours (Fig 2A and 2B). The accumulation of HMW-p62 in CORM-3-treated atg5-/- MEFs was also supported by immunocytochemistry: more intracellular p62-positive dots were observed in atg5-/- MEFs than in WT MEFs after CORM-3 exposure (Fig 2C). Taken together, the data show that CORM-3 (1 mM) causes the formation of HMW-p62. Since HMW-p62 accumulates in atg5-/- MEFs (Fig 2A), it seems likely that autophagy is involved in the elimination of this HMW-p62. Although more mono-p62 accumulated in atg5-/- MEFs than in WT, significantly higher HMW to mono p62 ratio was observed in atg5-/- MEFs than in WT (Fig 2B). This observation supports the notion that HMW-p62 should be eliminated through autophagy. We further examined HMW-p62 using non-reducing SDS-PAGE. CORM-3 treatment caused accumulation of aggregated p62, which could not enter the running gel, in atg5-/- MEFs (Fig 2D). Both aggregated and HMW-p62 were observed in both reducing and non-reducing SDS-PAGE, suggesting that aggregated p62 and HMW-p62 should be crosslinked through the formation of covalent bonds other than disulfide bonds (Fig 2D).

### Verteporfin generates HMW-p62 in MEFs

**Fig 3:**
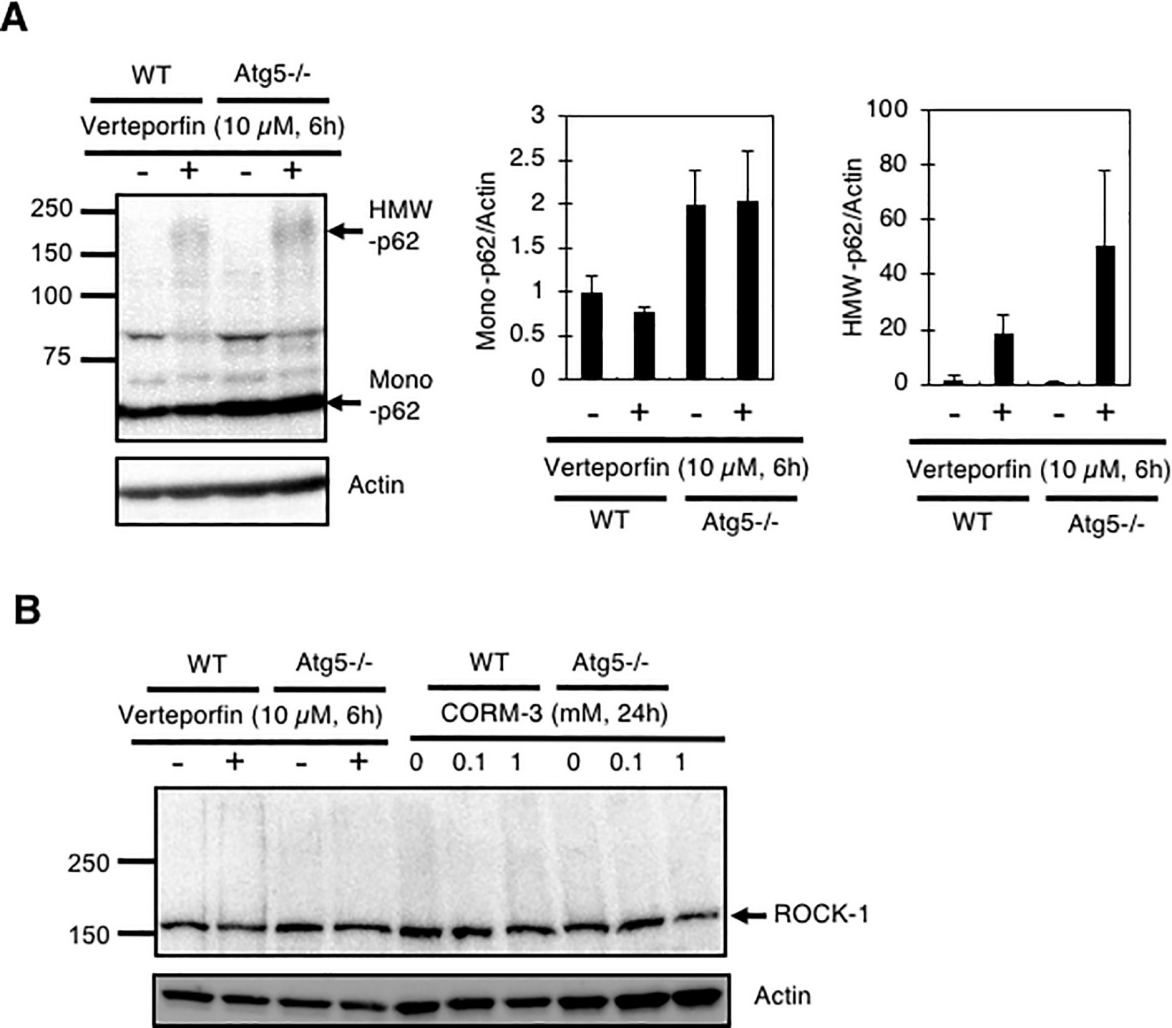
Generation of HMW-p62 by verteporfin in MEFs. (A) MEFs treated with 10 μM verteporfin for 6 hours were subjected to immunoblot analysis using anti-p62 antibody. Relative levels of p62 to actin are shown as means and S.E. (n = 4). (B) Lack of HMW-ROCK-1 in WT and atg5-/-MEFs treated with verteporfin or CORM-3. MEFs treated with 10 μM verteporfin for 6 hours or 1 mM CORM-3 for 24 hours were subjected to immunoblot analysis using anti-ROCK-1 antibody.

There are several chemicals, such as verteporfin [23], H_2_O_2_, [20], PR-619 [20], XIE62-1004 [21], and XIE2008 [21], which are reported inducing high molecular oligomers/aggregates of p62. It has also been reported that not only p62, but also other cellular proteins including ROCK-1 and Diap1 (Drosophila inhibitor of apoptosis protein-1), are covalently-crosslinked by verteporfin [27]. Therefore, we examined the effect of verteporfin on p62, as well as the effects of verteporfin and CORM-3 on ROCK-1, in MEFs. We observed HMW-p62 in MEFs exposed to verteporfin, confirming the previous report (Fig 3A) [23]. The apparent molecular weight of HMW-p62 generated by verteporfin treatment was somewhat different from that observed after CORM-3 treatment (~150 kDa by CORM-3 and ~200 kDa by verteporfin, Figs 2A and 3A), suggesting that HMW-p62 varies depending the chemical used. In contrast to the confirmation of the generation of HMW-p62 by verteporfin, we could not detect HMW-ROCK-1 in either verteporfin-treated or CORM-3-treated MEFs (Fig 3B). It has been reported that the protein-crosslinking ability of verteporfin is strongly enhanced under light [27]. Since we incubated the cells with verteporfin under dark conditions, one explanation for the differences involves light exposure.

### CORM-3 induces death in A549 and H9c2 cells accompanied by HMW-p62 formation

**Fig 4:**
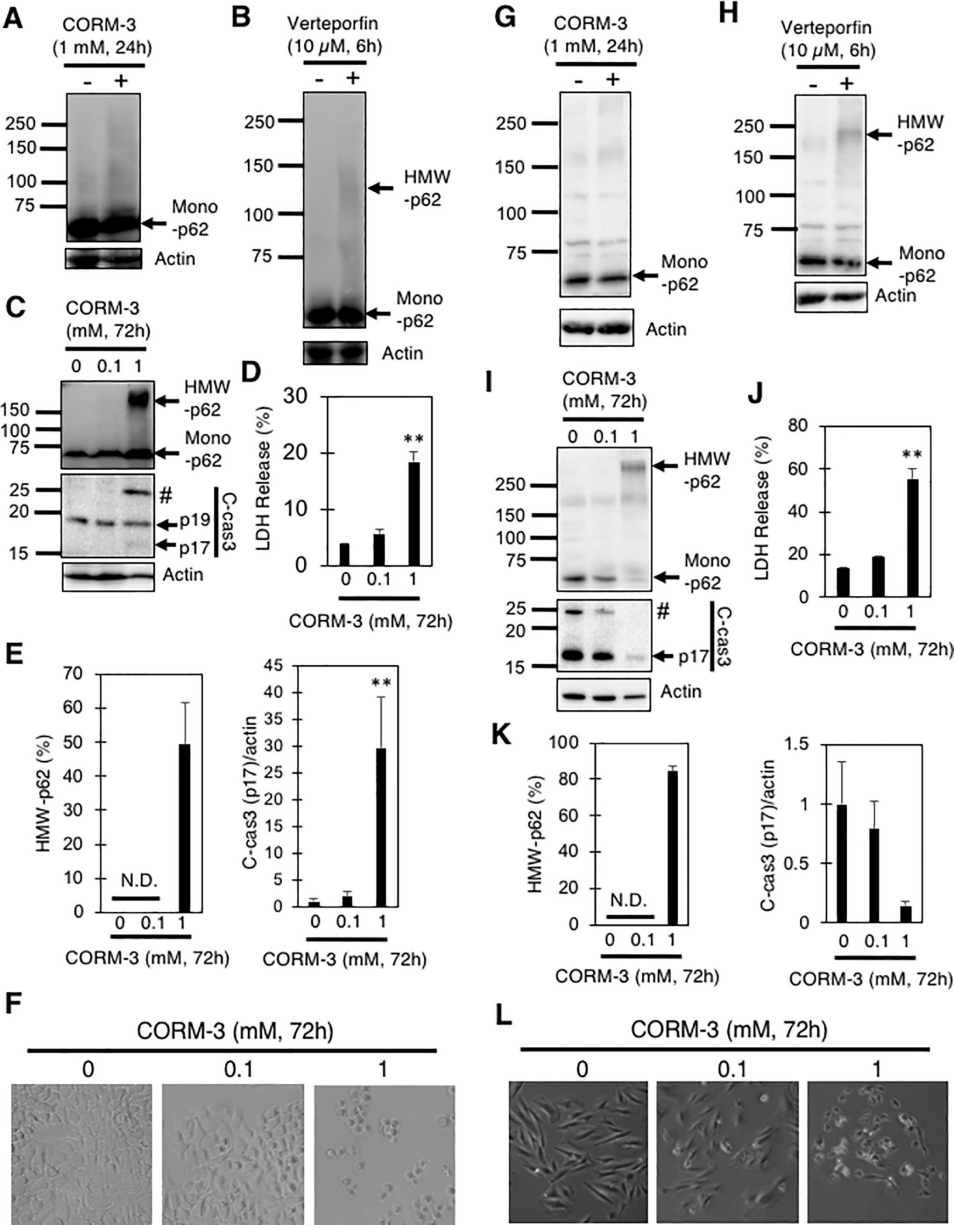
Generation of HMW-FN by CORM-3 in A549 and H9c2 cells. A549 cells (A-E) or H9c2 cells (G-K) treated with verteporfin (10 μM, 6 hours) or CORM-3 (0–1 mM, 24 or 72 hours) were subjected to immunoblot analysis using anti-p62 and anti-cleaved caspase 3 (c-cas3) antibodies. #, uncharacterized fragment. Percentages of HMW-p62 in total (HMW+mono) p62 and relative levels of c-cas3 (p17) to actin are also shown. (D and J) Percentages of LDH released from A549 (D) and H9c2 (J) cells into the medium 72 hours after treatment with the indicated concentrations of CORM-3. Graphs show means and S.E. (n = 4). **, p<0.01 versus control by Dunnett’s test. N.D., not detected. Representative phase contrast images of A549 (F) and H9c2 (L) cells treated with or without CORM-3 at the indicated concentrations for 72 hours are also shown.

We further examined whether HMW-p62 formation and cell death by CORM-3 are specific to MEFs or not. A549 cells derived from human lung and H9c2 cells derived from rat embryonic heart were used for this purpose. When A549 and H9c2 cells were treated with CORM-3 or verteporfin for the same time and at the same concentration used for MEFs, HMW-p62 was scarcely observed in either A549 or H9c2 cells (Fig 4A, 4B, 4G, and 4H). However, prolonged exposure to 1 mM CORM-3 (72 hours) resulted in the conversion of ~50% and ~80% p62 into HMW-p62 in A549 and H9c2 cells, respectively (Fig 4C, 4E, 4I, and 4K), demonstrating that HMW-p62 formation by CORM-3 is not specific to MEFs. Along with the formation of HMW-p62, c-cas3 (p17) levels were also increased in CORM-3-treated A549 cells, suggesting apoptotic cell death (Fig 4C and 4E). Curiously, the c-cas3 (p17) level was rather decreased in CORM-3-treated H9c2 cells (Fig 4I and 4K). LDH release assay confirmed that 15–20% of A549 cells (Fig 4D) and 50–60% of H9c2 cells (Fig 4J) died after treatment with 1 mM CORM-3 for 72 hours. Thus, although treatment with 1 mM CORM-3 for prolonged periods (72 hours) causes HMW-p62 formation and death in both A549 and H9c2 cells, the mode of cell death may vary depending on the cells examined.

### HMW-p62 formation by CORM-3 *in vitro*

**Fig 5:**
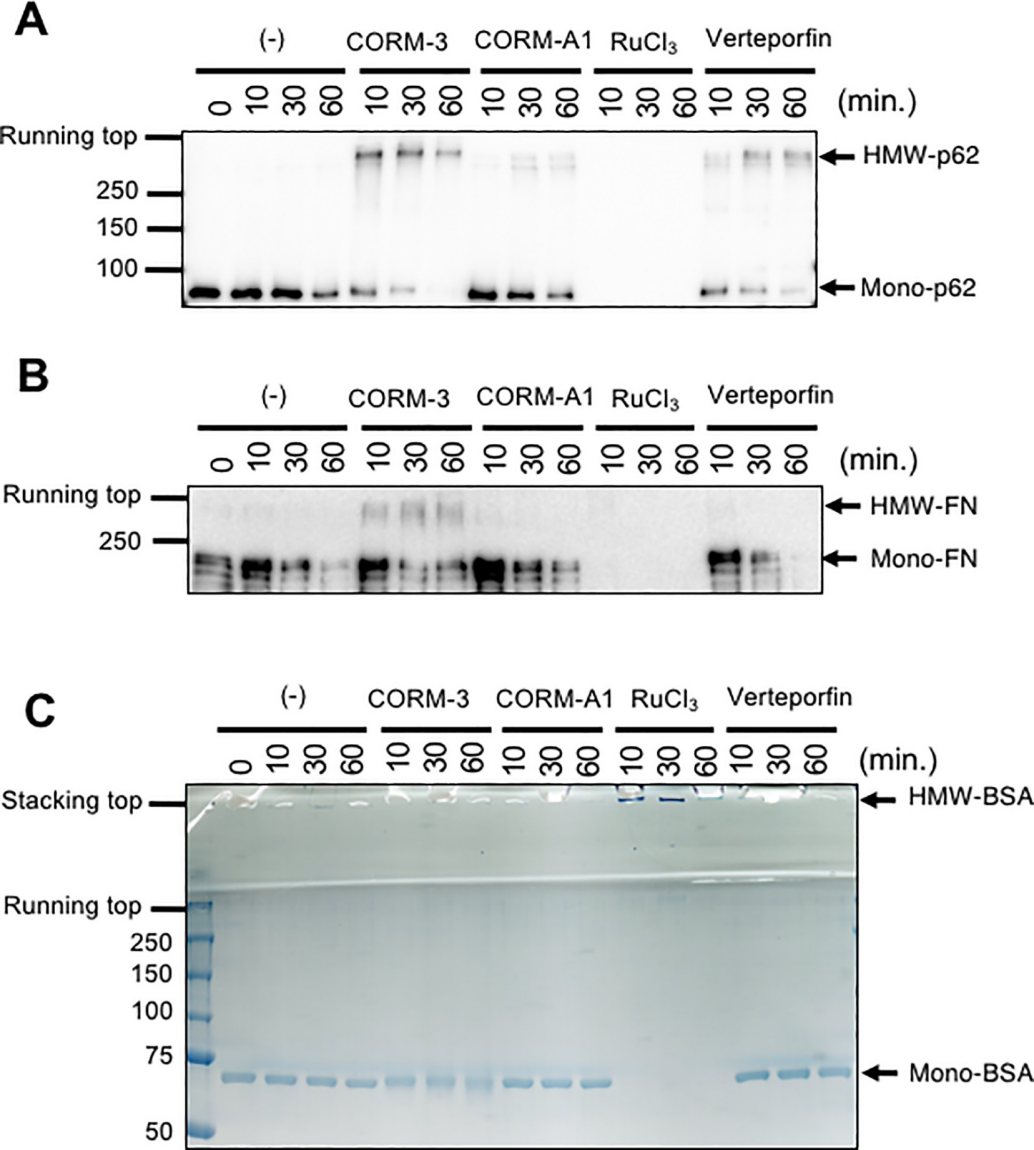
Production of HMW-p62 by CORM-3 *in vitro*. Purified recombinant full-length human p62 (A), bovine plasma fibronectin (FN) (B), or bovine serum albumin (BSA) (C) was dissolved in PBS and treated with CORM-3 (1 mM), CORM-A1 (1 mM), or RuCl_3_ (1 mM) for 0–60 min. p62, FN, and BSA were also treated with verteporfin (10 μM) for 0-60min. After SDS-PAGE, p62 and FN were visualized by immunoblot analysis while BSA was stained with CBB.

To investigate whether HMW-p62 is generated through a cellular process or results from the direct interaction between CORM-3 and p62, we examined whether HMW-p62 is formed when CORM-3 and p62 are incubated together *in vitro*. We previously reported that HMW-fibronectin (FN) formation by CORM-3 is dependent on the Ru ion rather than CO [15]. Thus, purified recombinant full length human p62 was incubated with 1 mM of CORM-3, CORM-A1 (non-metallic CORM), and RuCl_3_, as well as 10 μM of verteporfin, for 10–60 min. As shown in Fig 5A, CORM-3 rapidly (within 10 min.) induced the formation of HMW-p62 with an apparent molecular weight greater than 250 kDa. Purified plasma FN was also treated in the same manner as p62. The kinetics of HMW-p62 formation by CORM-3 resemble that of HMW-FN formation (Fig 5B). As demonstrated in our previous report, incubation of FN with RuCl_3_ *in vitro* resulted in the disappearance of FN (Fig 5B). Similar to FN, p62 also disappeared during incubation with RuCl_3_ (Fig 5A). During the treatment of p62 with CORM-A1, a slight but detectable formation of HMW-species was observed, suggesting that p62 might be more prone to aggregation than FN (Fig 5A and 5B). HMW-p62 was also created during incubation with verteporfin (Fig 5A), confirming the previous report [23]. In contrast, we could not detect HMW-FN after incubation with verteporfin (Fig 5B). Thus, there seems to be a preference for target proteins among HMW species-forming chemicals. We further examined the *in vitro* HMW formation assay using BSA, which has been shown to react with CORM-3 [28]. Incubation of BSA with CORM-3, CORM-A1, or verteporfin did not result in the formation HMW-BSA (Fig 5C), demonstrating that the HMW-species forming ability of CORM-3 should be specific to several proteins such as FN and p62. Interestingly, when BSA was incubated with RuCl_3_, we observed HMW-BSA accumulation at the top of stacking gel, demonstrating that this HMW-BSA was too large to enter the gels. Collectively, the *in vitro* HMW formation assay revealed that, like FN, p62 reacts with CORM-3 *in vitro*, and that HMW-p62 formation does not require cellular activity.

### Accumulation of Ub-positive aggregates associated with p62 in CORM-3-treated atg5-/- MEFs

**Fig 6:**
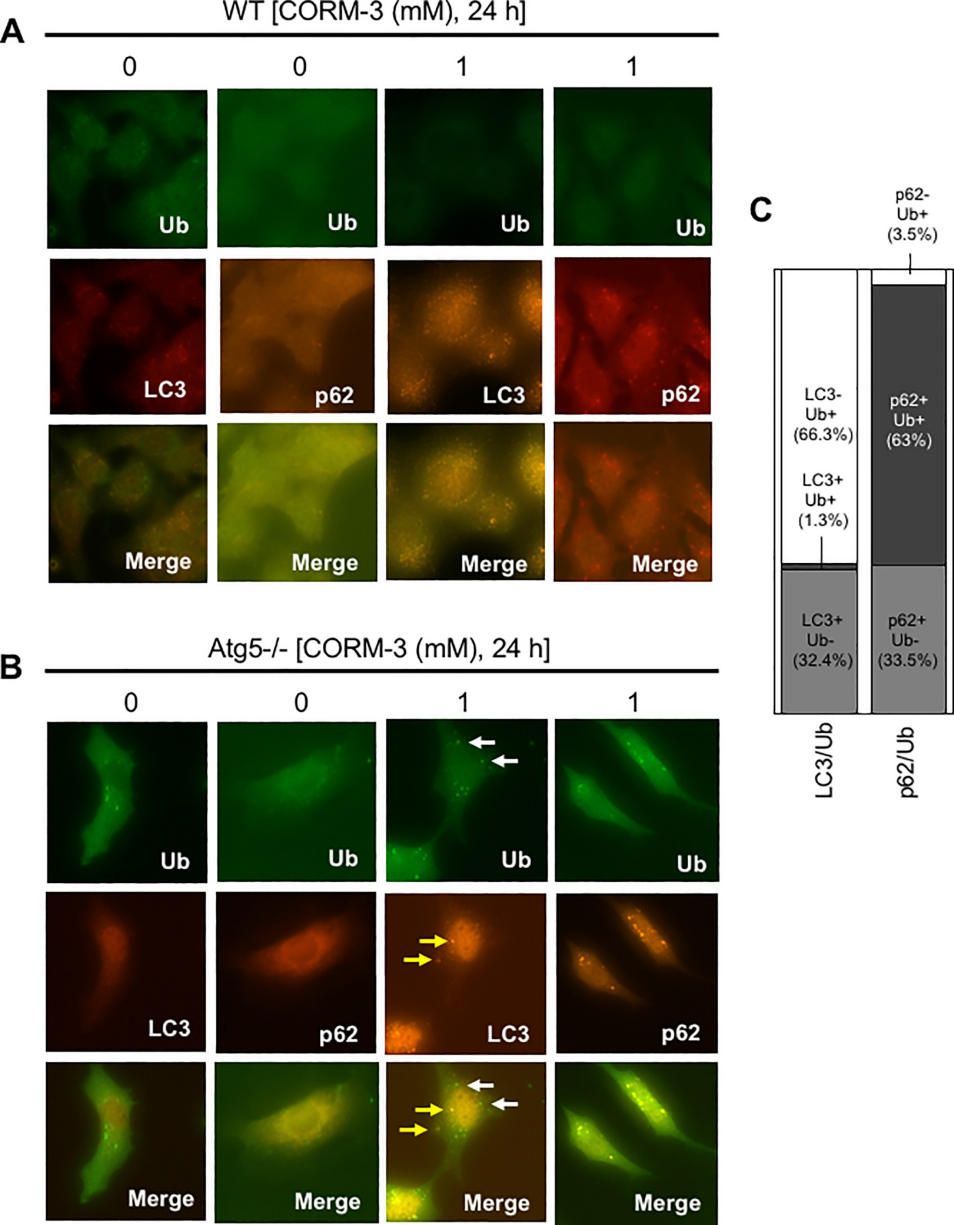
Co-localization of p62 dots with Ub-positive aggregates, but not with LC3-positive puncta, in atg5-/- MEFs. WT (A) and atg5-/- (B) MEFs were treated with or without 1 mM CORM-3 for 24 hours, and immunocytochemistry was performed using anti-ubiquitin conjugated protein (Ub), anti-p62, and anti-LC3 antibodies. Alexa488 (green)- and Alexa549 (red)-conjugated anti-IgG antibodies were used as secondary antibodies to visualize antigens under a fluorescence microscope. White and yellow arrows indicate Ub- and LC3-positive structures, respectively. Percentages of LC3-positive/Ub-negative (LC3+/Ub-), LC3-positive/Ub-positive (LC3+/Ub+), and LC3-negative/Ub-positive (LC3-/Ub+) dots, as well as p62-positive/Ub-negative (p62+/Ub-), p62-positive/Ub-positive (p62+/Ub+), and p62-negative/Ub-positive (p62-/Ub+) dots, in atg5-/- MEFs treated with 1 mM CORM-3 were shown (C).

Under physiological conditions, p62 binds to Ub-conjugated proteins and is incorporated into Ub-positive aggregates to facilitate their elimination through autophagy [17]. Therefore, we evaluated p62-positive, Ub-positive, and LC3-positive dots in CORM-3-treated MEFs by immunocytochemical analysis. Although Ub-positive dots were hardly observable in WT-MEFs regardless of CORM-3 treatment (Fig 6A), we did observe them in atg5-/- MEFs after treatment with CORM-3 (Fig 6B). In spite of the fact that atg5-/- MEFs do not produce autophagosomes, LC3 dots were observed in CORM-3-treated atg5-/- MEFs (Fig 6B). These LC3-positive dots should reflect the artificial aggregation of LC3 [29]. Indeed, LC3-positive dots did not merge with Ub-positive dots in atg5-/- MEFs treated with 1 mM CORM-3 (Fig 6B and 6C). In contrast, most of p62-positive dots were co-localized with Ub-positive dots, confirming the association of p62 with Ub-proteins (Fig 6B and 6C). These results imply that p62 undergoes aggregation together with Ub-proteins in CORM-3-treated atg5-/- MEFs.

### Knockdown of p62 ameliorates CORM-3-iuduced death of atg5-/- MEFs

**Fig 7:**
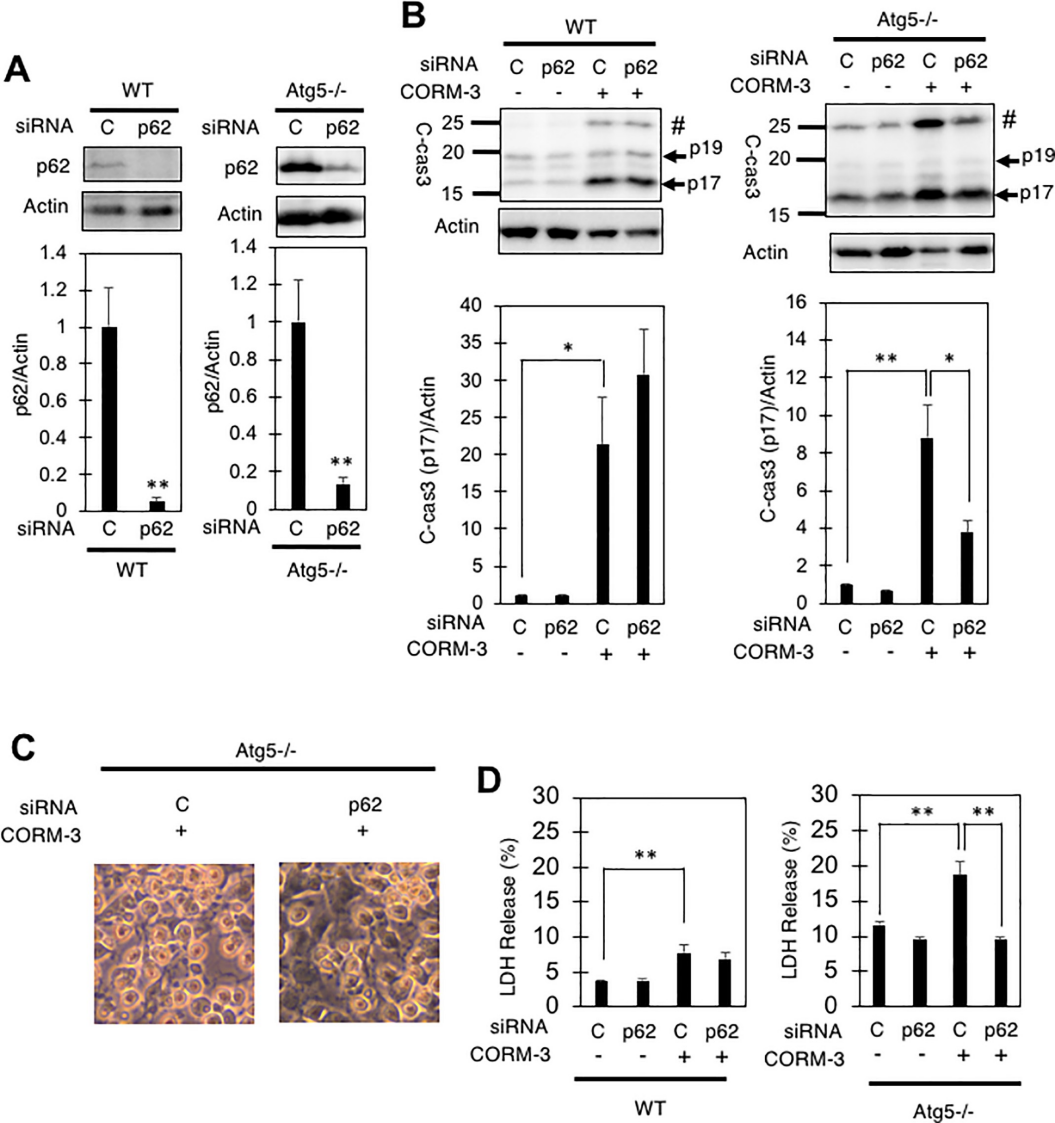
Effects of siRNA-mediated knock-down of p62 on CORM-3-induced death of atg5-/- MEFs. (A) WT and atg5-/- MEFs were treated with a control siRNA (C) or a siRNA for p62 (p62) and examined for their intracellular levels of p62 (left panels) by immunoblotting. #, uncharacterized fragment. The graphs show the means and S.E. of four samples. **, p<0.01 by student’s *t*-test. (B) SiRNA-treated MEFs were also exposed to 1 mM CORM-3 for 48 hours, and the relative levels of p17 fragment of cleaved-caspase-3 (c-cas3) were determined by immunoblotting. The graphs show the means and S.E. of four samples. *, p<0.05; **, p<0.01 by Turkey-Kramer’s test. Representative images of atg5-/- MEFs treated with or without the siRNA for p62 and then incubated with 1 mM CORM-3 for 48 hours, are shown (C). Percentages of LDH into the medium is also shown (D).

Finally, we examined whether p62 is involved in the cytotoxicity of 1 mM CORM-3 on MEFs. To this end, we evaluated the effects of small interference RNA (siRNA)-mediated knockdown of p62 on MEFs. Efficient knockdown of p62 by RNAi was confirmed in both WT and atg5-/- MEFs by immunoblotting (Fig 7A). Knock down of p62 by this siRNA resulted in a significant suppression of c-cas3 formation in CORM-3-treated atg5-/- MEFs (Fig 7B), which was also observed as a suppression of apoptosis by light microscopy (Fig 7C) as well as a suppression of LDH release (Fig 7D). These results suggest that p62 play detrimental role in atg5-/- MEFs during exposure to CORM-3.

## Discussion

In this study we provide evidence that CORM-3 causes HMW-p62 generation in MEFs, A549, and H9c2 cells. It has been shown recently that Ru(II) ion within CORM-3 react with several amino acids and a peptide, such as His, Met, Cys, and glutathione [30]. Indeed, CORM-3 covalently binds to a His residue within lysozyme [14]. Given the fact that CO is stable and scarcely react with other molecules except transition metals [2], CORM-3 reactivity toward p62 should be derived from Ru ion rather than CO.

To our knowledge, there are few reports describing HMW-p62 generation by chemicals. Verteporfin is one of the few chemicals inducing covalently crosslinked HMW-p62 formation [23]. p62 is comprised of several domains/regions, which include an N-terminal PB1 domain, an LC3-interacting region (LIR), and a C-terminal Ub-associated (UBA) domain [31]. The PB1 domain is considered to be responsible for p62 oligomerization both through covalent [21, 23] and non-covalent interactions [18, 32]. Thus, PB1 domain is most likely to be responsible for HMW-p62 formation by CORM-3. Indeed, a mutant of p62, which possesses K7A/D69A mutations within PB1 domain, has been shown to lose the ability to form HMW-p62 in response to verteporfin treatment [23]. Since these two residues (K7 and D69) are involved in the physiological oligomerization through homotypic electrostatic interaction between p62 [32], HMW-p62 formation by CORM-3 might also be dependent on non-covalent interactions between p62.

Although verteporfin inhibits autophagy through the formation of HMW-p62, CORM-3 does not seem to inhibit autophagy as judged by LC3-II formation (Fig 2A). It has been reported that HMW-p62 generated by verteporfin retains the ability to bind to LC3 although it has less ability than non-covalently crosslinked p62 to bind to Ub-proteins [23]. In contrast, HMW-p62 generated by CORM-3 seems to bind to Ub-proteins as judged by the results of immunocytochemistry (Fig 6B and 6C). However, more comprehensive experiments are necessary to elucidate whether HMW-p62 generated by CORM-3 retains its Ub-protein and/or LC3-binding ability or not.

Recently, Carroll *et al*. reported p62 disulfide-linked conjugates (DLC) [20]. p62-DLC accumulates during aging in mammals, and facilitates autophagy in response to oxidative stresses. Interestingly, p62-DLC formation does not require non-covalent interaction between PB1 domain [20]. However, HMW-p62 and p62-DLC are clearly different from each other based on the following observations. First, p62-DLC results from the formation of disulfide bonds between cysteine residues, and, therefore, resolves into monomers under reducing conditions [20]. In contrast, HMW-p62 is observed even under reducing conditions (Figs 2A, 3A, and 5A). Also, p62-DLC facilitates autophagy [20], whereas HMW-p62 generated by verteporfin has been shown to inhibit autophagy [23] and HMW-p62 generated by CORM-3 seems not to be involved in the autophagic process, at least at the step of LC3-II formation (Fig 2A).

Although CORM-3 was designed as a CO-releasing molecule, recent research has indicated that many of the effects of CORM-3 on eukaryotic as well as prokaryotic cells are derived from Ru ion rather than CO [30, 33]. In accordance with this notion, we observed the formation of HMW-BSA, which cannot enter the gel during SDS-PAGE, by RuCl_3_ (Fig 5C). Thus, the disappearance of p62 and FN after incubation with RuCl_3_ during SDS-PAGE (Fig 5A and 5B) might indicate that p62 and FN also undergo extensive self-oligomerization during RuCl_3_ treatment so as not to enter the gel. We tried to detect HMW-p62 and -FN by blotting whole parts of the gel (including the stacking and running gels) to PVDF membranes, but failed to detect HMW-p62 and -FN in RuCl_3_ treated samples (data not shown). This might indicate that HMW-p62 and -FN are unstable during the immunoblotting procedure if they remained on top of the stacking gel. Alternatively, unlike HMW-BSA, HMW-p62 and -FN might undergo extensive degradation in response to RuCl_3_ treatment.

In conclusion, we have shown in this study an example in which a high concentration (1 mM) of CORM-3 causes protein oligomerization even of an intracellular protein. This study provides a new viewpoint for understanding the intracellular actions of metallic CO-releasing molecules on mammalian cells.
